# Cooperative therapeutic anti-tumor effect of IL-15 agonist ALT-803 and co-targeting soluble NKG2D ligand sMIC

**DOI:** 10.18632/oncotarget.6416

**Published:** 2015-11-27

**Authors:** Fahmin Basher, Emily K. Jeng, Hing Wong, Jennifer Wu

**Affiliations:** ^1^ Department of Microbiology and Immunology, Medical University of South Carolina, Charleston, SC, USA; ^2^ Altor BioSciences Corporation, Miramar, FL, USA; ^3^ Cancer Immunology Program, Hollings Cancer Center, Charleston, SC, USA; ^4^ CanCure LLC, Everett, WA, USA

**Keywords:** ALT-803, soluble NKG2D, therapy, tumor

## Abstract

Shedding of the human NKG2D ligand MIC (MHC class I-chain-related molecule) from tumor cell surfaces correlates with progression of many epithelial cancers. Shedding-derived soluble MIC (sMIC) enables tumor immune escape through multiple immune suppressive mechanisms, such as disturbing natural killer (NK) cell homeostatic maintenance, impairing NKG2D expression on NK cells and effector T cells, and facilitating the expansion of arginase I^+^ myeloid suppressor cells. Our recent study has demonstrated that sMIC is an effective cancer therapeutic target. Whether targeting tumor-derived sMIC would enhance current active immunotherapy is not known. Here, we determined the *in vivo* therapeutic effect of an antibody co-targeting sMIC with the immunostimulatory IL-15 superagonist complex, ALT-803, using genetically engineered transplantable syngeneic sMIC^+^ tumor models. We demonstrate that combined therapy of a nonblocking antibody neutralizing sMIC and ALT-803 improved the survival of animals bearing sMIC^+^ tumors in comparison to monotherapy. We further demonstrate that the enhanced therapeutic effect with combined therapy is through concurrent augmentation of NK and CD8 T cell anti-tumor responses. In particular, expression of activation-induced surface molecules and increased functional potential by cytokine secretion are improved greatly by the administration of combined therapy. Depletion of NK cells abolished the cooperative therapeutic effect. Our findings suggest that administration of the sMIC-neutralizing antibody can enhance the anti-tumor effects of ALT-803. With ALT-803 currently in clinical trials to treat progressive solid tumors, the majority of which are sMIC^+^, our findings provide a rationale for co-targeting sMIC to enhance the therapeutic efficacy of ALT-803 or other IL-15 agonists.

## INTRODUCTION

Ligand-induced activation of the NK cell activating receptor NKG2D has been demonstrated to be significant in controlling tumor growth in experimental animal models [1, 2]. NKG2D, an NK cell group 2 member D activating receptor, is also expressed by most NKT cells, subsets of gamma-delta T cells, all human CD8^+^ T cells, and activated mouse CD8^+^ T cells as co-stimulatory receptors [1, 3–7]. Ectopic expression of NKG2D ligands results in effective tumor rejection mediated by NK cells, and in some cases primed cytotoxic T cells [5, 6]. Neutralization of NKG2D was shown to increase the incidence of carcinogen-induced tumor formation [8]. In the spontaneous prostate tumor model TRAMP (transgenic adenocarcinoma of the mouse prostate), mice deficient in NKG2D exhibited accelerated tumor progression in comparison to their NKG2D^wt^ counterparts [9]. Enforced expression of membrane-bound NKG2D ligands on TRAMP tumors prevented disease progression through sustained NKG2D signaling in effector cells [10]. These studies have collectively demonstrated the importance of NKG2D signaling in active tumor immune surveillance.

The most prevalently expressed NKG2D ligands in human malignancies are the MHC class I-related molecules A and B (MICA/B, collectively termed MIC) [11–14]. However, human cancer cells frequently evade NKG2D signaling through protease or exosome-mediated shedding of MIC to produce the immune suppressive soluble MIC (sMIC). Clinically, levels of sMIC correlated with disease stage in many malignancies [10, 15–19]. Mechanistically, sMIC shed from tumor cells has been shown to down-modulate surface NKG2D expression on NK cells and effector NKT and T cells [17, 20, 21]. Very recently, we have further shown that sMIC perturbs NK cell homeostatic maintenance in tumor-bearing hosts and facilitates the expansion of arginase I^+^ myeloid suppressor cells in the tumor microenvironment [10, 22]. These studies suggest that sMIC may be a valid target for immunotherapy to re-instate endogenous NK and effector T cell anti-tumor immune responses. Our recent study has provided proof for this concept [14].

The common gamma-chain cytokine interleukin-15 (IL-15) is considered as one of the most promising cytokines for cancer immunotherapy [23–27]. IL-15 not only is important for maintaining NK cell homeostasis [28–33], but also can upregulate NKG2D expression on NK and T cells and prime NKG2D signaling pathways [34–37]. IL-15 has also been shown to be important for maintaining CD8^+^ memory T cell populations [38–42]. The IL-15 superagonist ALT-803, a complex of the mutant IL-15 (IL-15N72D) and the IL-15RαSu/Fc [43, 44], was shown to confer at least 25-fold higher bioactivity *in vivo* and extended half-life compared to native IL-15 [45]. Pre-clinical studies have demonstrated that a single dose of ALT-803 was able to eliminate well-established primary myeloma cells in the bone marrow and to further reject tumor re-challenge due to expansion of CD44^hi^ memory CD8^+^ T cells [45]. These pre-clinical studies have signified the cancer therapeutic potential of ALT-803 and have led to the current clinical trials for treating various human malignancies [46]. However, due to the facts that mice do not express human MIC and the human onco-immune dynamics of NKG2D ligand shedding and tumor progression have not been described in these mouse models, the impact of tumor-derived immune suppressive sMIC on the therapeutic potential of ALT-803 remains unknown.

To overcome the limitation that mice do not express human MIC, we have developed syngeneic transplantable tumor models in which sMIC-overexpressing mouse tumor cell lines were implanted into the sMIC-tolerant transgenic mouse [10]. Using this transplantable system, we tested the hypothesis that ALT-803 and a sMIC-neutralizing antibody can generate a cooperative therapeutic anti-tumor effect. We demonstrate that combinatory therapy of an antibody targeting sMIC and ALT-803 significantly enhanced the survival of mice bearing sMIC^+^ tumors in comparison with monotherapy. Mechanistically, we show that combined therapy cooperatively enhanced the homeostatic maintenance and functional potential of NK cells and memory CD8^+^ T cells. Combinatory therapy also heightened the potential of CD4^+^ T cells to produce IFN-γ and cooperatively eliminated myeloid derived suppressor cells (MDSCs) in tumor infiltrates. We also demonstrate *in vitro* that ALT-803 and a sMIC-neutralizing antibody cooperatively enhanced the activation of STAT5 signaling pathways in effector cells. Our findings provide the rationale for a translational approach whereby combinatory therapy of an antibody targeting tumor-derived sMIC and ALT-803 can cooperatively enhance innate and adaptive anti-tumor responses.

## RESULTS

### ALT-803 and sMIC-neutralizing antibody combined therapy inhibits tumor growth and prolongs survival of animals bearing sMIC^+^ tumors

Tumor shedding of sMIC is a human-specific mechanism of tumor immunoevasion. To test the hypothesis that targeting sMIC can enhance the therapeutic potential of IL-15 superagonist ALT-803 in a pre-clinical model, we developed multiple transplantable syngeneic tumor models by: 1) overexpressing human soluble MICB in transplantable mouse tumor cell lines, and 2) inoculating tumor lines secreting sMICB into the MICB transgenic mouse. As membrane-bound MIC can stimulate anti-tumor immunity [10], in order to eliminate experimental variation, we chose to develop these tumor models using the soluble form of MICB instead of membrane-bound MIC. Since mice do not express homologs of the human MIC ligand family, we utilized MICB transgenic mice as hosts to eliminate the effect of autoantibodies against the human sMICB. The MICB transgenic mice were produced by using the minimal rat probasin (rPb) promoter to direct expression of the transgene encoding the native form of MICB to the prostate epithelium. These mice have a similar phenotype as wild type B6 animals; however, they do not generate immune responses to syngeneic tumors expressing human MIC [10].

**Figure 1:**
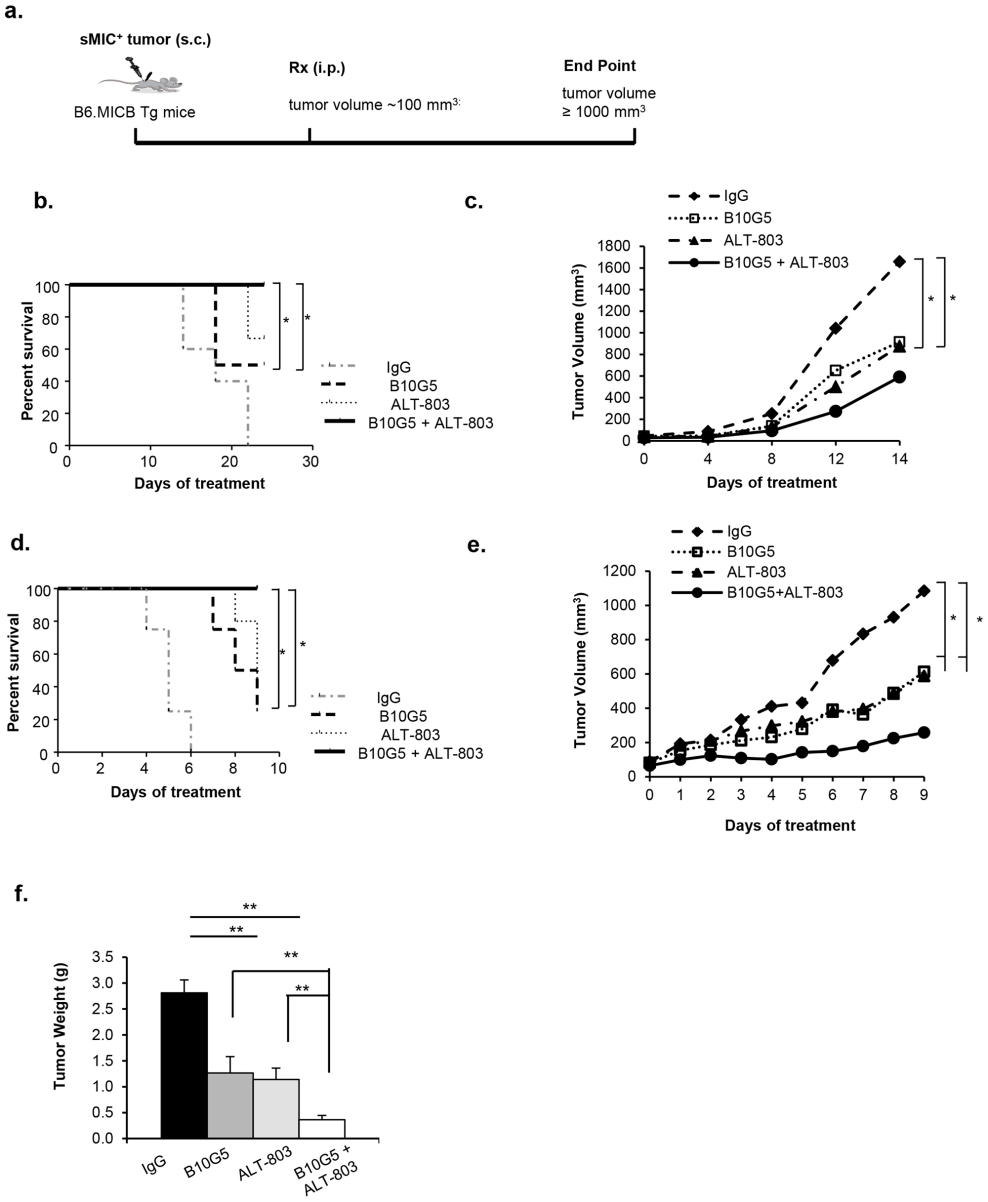
Combined therapy inhibits tumor growth and prolongs survival of animals bearing sMIC^+^ tumors **a.** Depiction of treatment scheme. B6/MICB mice received right flank subcutaneous injection of 4 × 10^6^ RM9-sMICB or B16F10-sMICB cells were treated i.p. with: 1) control mIgG (*n* = 10), 2) B10G5 (*n* = 9), 3) IL-15 superagonist ALT-803 (*n* = 8), and 4) a combination of B10G5 and ALT-803 (*n* = 10). **b.** Survival curve of mice bearing RM9-sMICB tumors with different therapy. **c.** Growth curve of RM9-sMICB tumors under different therapies. **d.** Survival curve of mice bearing B16-sMICB tumors with different therapy. **e.** and **f.** Growth curve of B16-sMICB tumors during different therapies. (f) Total weights of B16-sMICB tumors when animals were sacrificed at day 10 of therapy in repeated experiments. *n* = 8–10 per experimental group. *p < 0.05. **p < 0.01.

We implanted the murine mouse prostate tumor cell line RM9 and melanoma cell line B16F10 that were engineered to express human sMICB (designated as RM9-sMICB and B16-sMICB respectively) subcutaneously into cohorts of syngeneic MICB transgenic mice. When tumors reached approximately 75–100 mm^3^ in volume, mice were randomized into four therapeutic groups (*n* = 8–10 per group, Figure 1a). Although monotherapy with the sMIC-neutralizing antibody B10G5 and ALT-803 elicited survival benefits in comparison to control treatment, combined therapy further significantly prolonged survival in comparison to monotherapy in two independent tumor transplants (*p* < 0.05 and *p* < 0.0001 respectively, Figure 1b and 1d). Using linear regression analyses, we compared tumor growth rate prior to animals in the control group (or any treatment group) reaching the survival endpoint. B10G5 or ALT-803 monotherapy significantly reduced tumor growth rate by 35% and 51%, respectively, in comparison to control IgG treatment, whereas combined therapy further reduced tumor growth rate by 60% (Figure 1c). We observed similar trends in mice bearing B16-sMICB tumors (Figure 1d). Monotherapy with either B10G5 or ALT-803 reduced the average tumor burden by about 50%, whereas combined therapy further reduced tumor burden by more than 70% (*p* < 0.001) (Figure 1e). The enhanced therapeutic effect of combined therapy was further confirmed by the significant reduction in final B16-sMICB tumor weight in comparison to monotherapy in repeated experiments in which all animals were euthanized at day 10 of therapy (*p* < 0.01) (Figure 1f). Similar trend in survival benefit and tumor growth rate with combined therapy in comparison to monotherapy was presented with treatment up to 55 days where majority (>90%) of animals reached defined survival end point (data not shown). These data clearly demonstrate that monotherapy of ALT-803 or the sMIC-neutralizing antibodyB10G5 has respective therapeutic effects; however combined therapy elicited significantly enhanced therapeutic effects.

### ALT-803 and sMIC-neutralizing antibody combined therapy markedly enhances NK cell number and function

**Figure 2:**
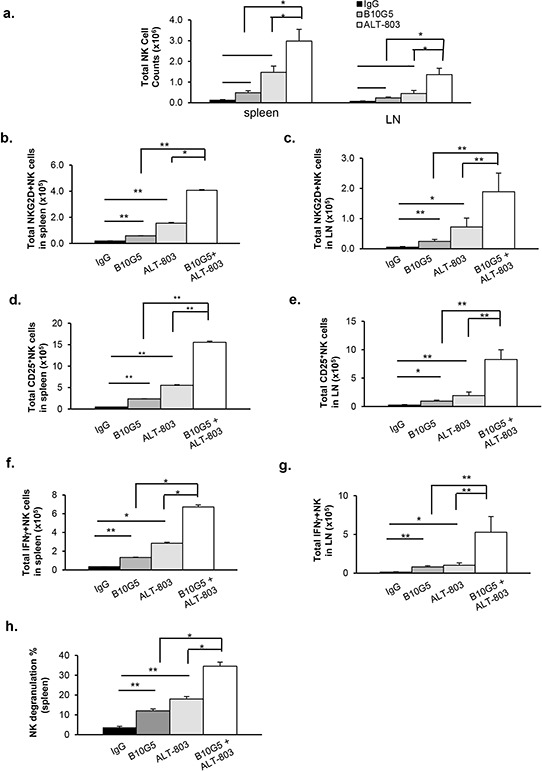
ALT-803 and sMIC-neutralizing antibody combined therapy markedly enhances NK cell homeostatic maintenance and function in sMIC-B16 tumor bearing mice **a.** Therapy significantly increased total number of NK (CD3^−^NK1.1^+^) cells in the spleen or tumor-draining lymph nodes (dLN). **b, c.** Therapy significantly increased total number of NKG2D^+^ NK cells in the spleen and dLN. **d, e.** Therapy significantly increased NK cell activation shown by increased population of CD25^+^NK cells. **f-i.** Therapy significantly enhanced NK cell function shown by IFNγ production in response to PMA/Ionomycin *in vitro* re-stimulation. f and g, summary data of total IFNγ^+^NK cells. h. Summary of splenic NK cell cytotoxic function shown by CD107a degranulation when co-cultured with target RMA-S-RAE-1β cells (4:1 ratio). In all criteria, combined therapy elicited significantly better response than monotherapy. Similar results were obtained from sMIC-RM9 tumor bearing mice. *p < 0.05. **p < 0.01. Groups with statistically non-significances were not marked.

We sought to understand mechanisms associated with the enhanced efficacy of combined therapy. Tumor-derived soluble MIC not only impairs NK cell function by downmodulating surface NKG2D expression but also perturbs peripheral NK cell maintenance [10, 21]. We first investigated the therapeutic effect of monotherapy and combined therapy on NK cells. As representatively shown with the B16-sMICB tumor model, in response to monotherapy with B10G5 or ALT-803, not only the number of NK cells (CD3^−^NK1.1^+^) but also the level of NKG2D expression, measured by the percentage of NKG2D^+^ NK cells and the mean fluorescence intensity (MFI) of the NKG2D expression on NKG2D^+^ NK cells, in the spleen and tumor-draining inguinal lymph nodes (dLN) was markedly increased in comparison to the control treatment group (Figure 2a–2c and Supplementary Figure S1a). However, combined therapy resulted in further significant increase in the number of NK cells and the level of NKG2D expression on cells in the spleen and dLN compared to monotherapy (Figure 2a–2c; Supplementary Figure S1a). Moreover, significantly increased activation of NK cells as evaluated by surface CD25 expression, was also observed in response to therapy, either by monotherapy of B10G5 or ALT-803(Figure 2d and 2e; Supplementary Figure S1b). A further significant increase in surface CD25 expression on NK cells was elicited with combination therapy in comparison to monotherapy (Figure 2d and 2e; Supplementary Figure S1b), either with B10G5 or ALT-803, indicating enhanced NK cell activation with combination treatment. No significant change in other NK cell surface receptor expression, such as NKp46, Ly49A, Ly49C/I/F/H, NKG2A/C/E, was observed with combined therapy in comparison to monotherapy (data not shown).

We further tested functional potential of NK cells from these mice by responses to *ex vivo* re-stimulation. NK cell IFNγ production in response to *ex vivo* PMA/I stimulation or degranulation shown by CD107a expression in response to NKG2D ligand-positive RMA-S-RAE-1β stimulation was significantly increased with monotherapy of B10G5 or ALT-803 (Figure 2f–2h). When B10G5 and ALT-803 were combined, therapy resulted in a further significant increase in the number of IFNγ-producing NK cells in response to PMA/I stimulation and the level of NK cell degranulation in response to RMA-S-RAE1β stimulation (Figure 2f–2h, Supplementary Figure 1c and 1d). These data clearly demonstrate that, in comparison to monotherapy, combined therapy significantly enhanced innate NK cell anti-tumor potential by further restoring homeostatic maintenance and function.

### Combined therapy enhances functional potential of CD8 T cells

**Figure 3:**
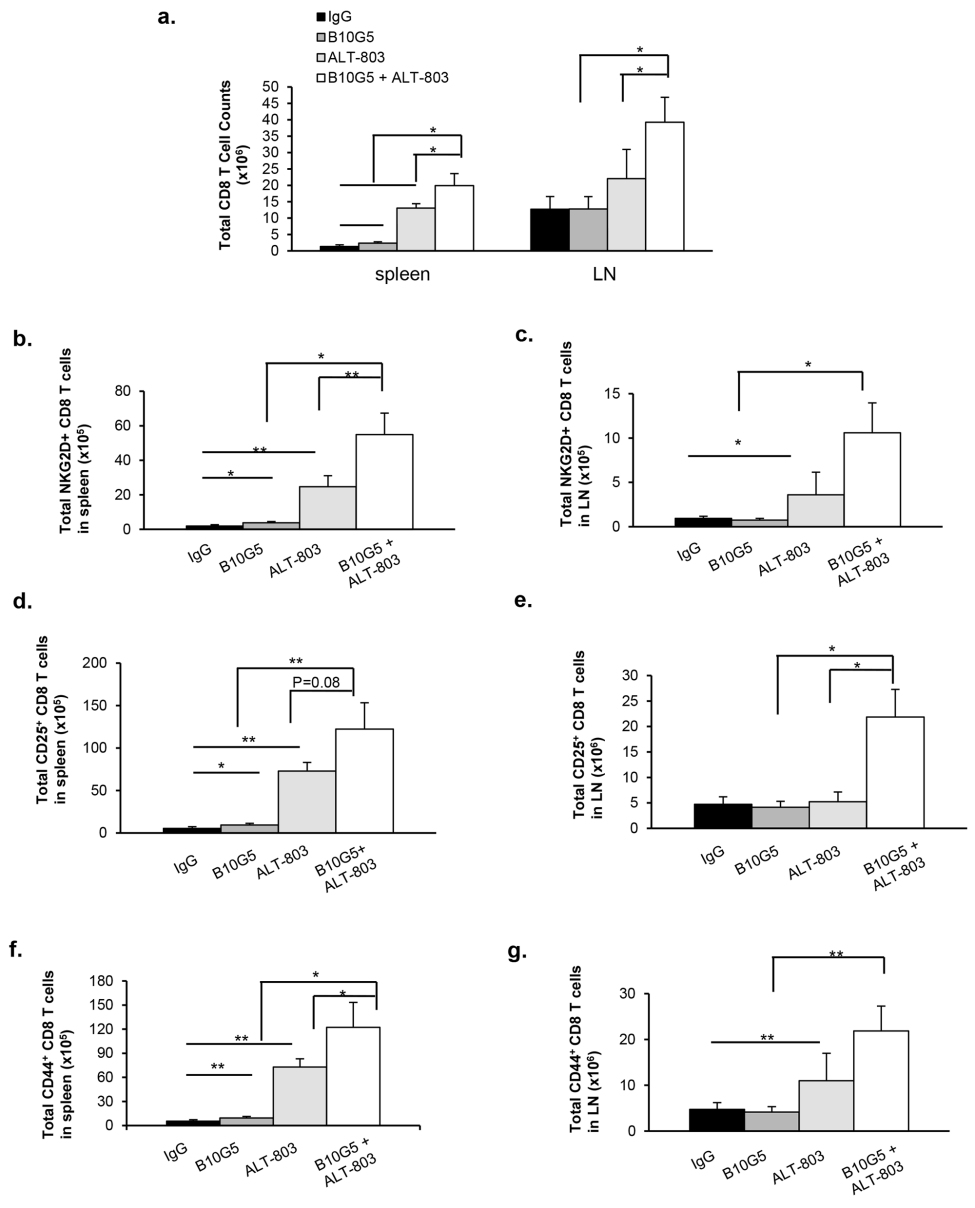

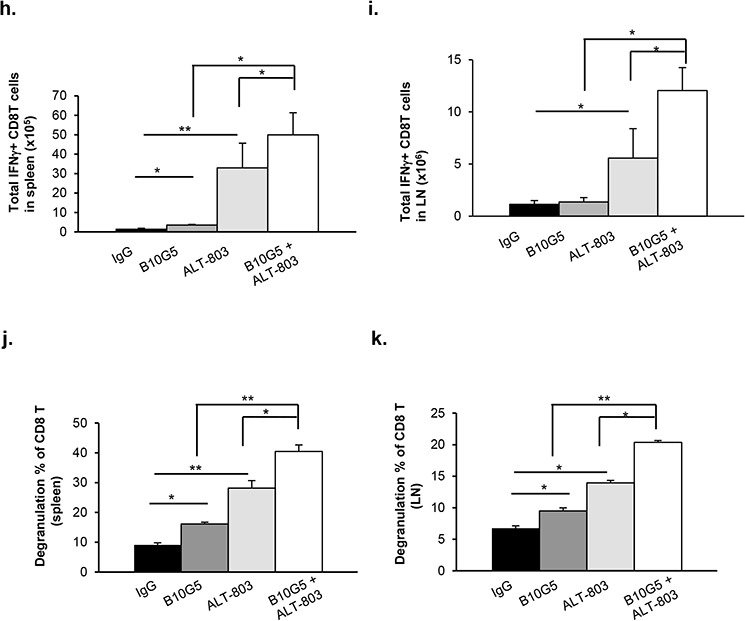
Combined therapy remarkably enhanced functional potential of CD8 T cells in sMICB-B16 tumor bearing mice **a.** Combined therapy significantly increased total number of CD8 T cells in the spleen and lymph node. **b, c.** Combined therapy remarkably increased NKG2D^+^CD8 T cell populations in the spleen and dLN. **d, e.** Combined therapy significantly increased the number CD25^+^ CD8 T cells in the spleen and dLN, whereas monotherapy only elicited limited effect. **f, g.** Combined therapy significantly increased CD44^+^ CD8 T cell population, whereas monotherapy only elicited limited effect. **h, i.** Combined therapy significantly increased CD8 T cell anti-tumor potential shown by IFNγ-production in response to *in vitro* PMA/Ionomycin re-stimulation; whereas monotherapy only elicited limited effect. Similar results were obtained from sMIC-RM9 tumor bearing mice. **j, k.** Combined therapy significantly increased CD8 T cell cytotoxic potential shown by CD107a degranulation in response to tumor peptide antigen gp100 stimulation. Data were obtained at day 10 since treatment initiation. *p < 0.05. **p < 0.01. Groups with statistically non-significances were not marked.

We next sought to determine the effects of combined therapy on adaptive immunity, primarily CD8^+^ T cells, as ALT-803 has been demonstrated to enhance CD8^+^ populations *in vivo* [48]. As shown in Figure 3a, in mice bearing B16-sMICB tumors, monotherapy of B10G5 or ALT-803 increased splenic CD8 numbers by 75% and 860%, respectively, compared to control treatment. With combined treatment, splenic CD8 numbers further increased significantly compared to monotherapy with B10G5 (*p* < 0.005) or ALT-803 (*p* = 0.05) (Figure 3a). The frequency of NKG2D^+^CD8^+^ T cells was significantly increased in both the spleen and dLN with monotherapy of B10G5 or ALT-803 compared to control IgG therapy (Figure 3b and 3c, Supplementary Figure S2a). With combined treatment, splenic NKG2D^+^CD8^+^ T cell populations and the intensity of NKG2D expression (measured by MFI) on NKG2D^+^CD8^+^ T cell were further markedly increased in comparison to monotherapy of B10G5 (*p* < 0.05) or ALT-803 (*p* < 0.01) (Figure 3b and 3c; Supplementary Figure S2a). Since NKG2D is only expressed by activated CD8^+^ T cells in mice [49], these data indicate a cooperation of B10G5 and ALT-803 in activating CD8^+^ T cells. This conclusion was further confirmed by examination of CD25^+^ CD8^+^ T cells populations (Figure 3d and 3e; Supplementary Figure S2b).

Previous studies have shown that ALT-803 can increase CD44^hi^ memory CD8^+^ T cell populations in tumor-bearing hosts [45]. Consistent with these studies, monotherapy with ALT-803 increased the population of memory-like CD44^hi^ CD8^+^ T cells in both the spleen and dLN. B10G5 alone resulted in a significant increase in CD44^hi^ CD8^+^ T cells in the spleen, but not in the dLN. Intriguingly, a remarkable increase in the population of CD44^hi^ CD8^+^ T cells in both spleen and dLN was elicited as a result of the combined therapy (Figure 3f and 3g; Supplementary Figure S2c). These data demonstrate the cooperative effect of ALT-803 and B10G5 in generating or likelihood maintaining memory CD8^+^ T cells in the tumor-bearing host.

IL-15 has been shown to revive CD8^+^ T cells from an anergic state in tumor hosts [50–52]. sMIC has been shown to impair NKG2D expression and thus the function of CD8^+^ T cells [20]. These findings prompted us to evaluate whether there is a combined therapeutic effect on CD8^+^ T cell function. Consistent with other studies, ALT-803 therapy significantly increased the frequency and total number of splenic IFN-γ-producing CD8^+^ T cells in response to PMA and ionomycin re-stimulation *in vitro* (Figure 3h and 3i; Supplementary Figure S2d). To a lesser extent than ALT-803, B10G5 therapy also significantly increased the number of splenic IFN-γ-producing and CD8^+^ T cells. With the combined therapy of ALT-803 and B10G5, CD8^+^ T cells elicited a markedly enhanced response to re-stimulation in comparison to monotherapy (Figure 3h and 3i, Supplementary Figure S2d). Moreover, with combined therapy, CD8 T cells exhibited greater cytotoxic responses shown by increased CD107a degranulation with melanoma peptide antigen gp100 stimulation (Figure 3j and 3k, Supplementary Figure S2e). Together, these data have suggested that combined therapy of B10G5 and ALT-803 cooperatively not only can improve the generally machinery responsiveness of CD8 T cells in sMIC^+^ tumor-bearing mice, but also can enhance antigen-specific effector CD8 T cell responses.

### Combination therapy of B10G5 and ALT-803 heightens CD4^+^ T cell anti-tumor potential

**Figure 4:**
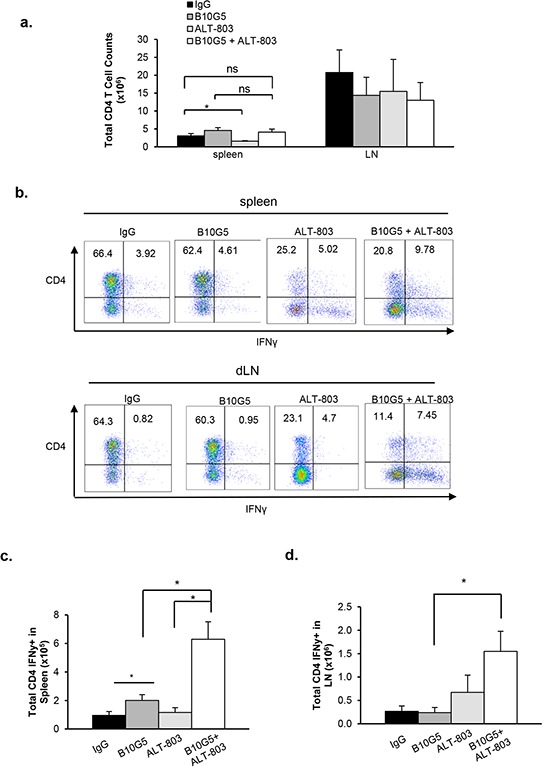
Combinatory therapy of B10G5 and ALT-803 significantly heightens CD4 T cell anti-tumor potential **a.** Total number of CD4 T cells remains unchanged in the spleen and dLN in response to therapy, whether monotherapy or combined therapy. **b.** Representative dot plots demonstrating the impact of therapy on the frequency of IFNγ-producing CD4 T cells in the spleen and dLN in response to *in vitro* PMA/Ionomycin re-stimulation. **c.** Summary data showing the total number of IFNγ-producing CD4 T cells in response to therapy. Data were obtained at day 10 since treatment initiation. Similar results were obtained from sMIC-RM9 tumor bearing mice. *p < 0.05.

IL-15 has been shown to be important in driving TCR-dependent expansion of CD4^+^ T cells and maintaining memory function [53–56]. Thus, we sought to determine our combined therapeutic effect on CD4^+^ T cells. ALT-803 monotherapy had a trend of reduced total CD4 T cell population in the spleen (*p* = 0.049), however, combined therapy did not significantly impact total CD4 numbers in the spleen (Figure 4a). Neither monotherapy with B10G5 or ALT-803 nor combined therapy had a significant impact on total CD4^+^ T cell numbers in tumor-draining LNs (Figure 4a). Monotherapy of B10G5 or ALT-803 had differential impact on potentiating CD4^+^ T cell to Th1 responses in the spleen and dLN. As shown by IFNγ production in response to *in vitro* PMA/ionomycin re-stimulation, B10G5 therapy resulted in a better Th1 response in the spleen, whereas ALT-803 therapy remarkably potentiated CD4^+^ T cells to Th1 responses in the spleen and dLN (Figure 4b–4d).

### B10G5 and ALT-803 reduce MDSC populations in TILs

**Figure 5:**
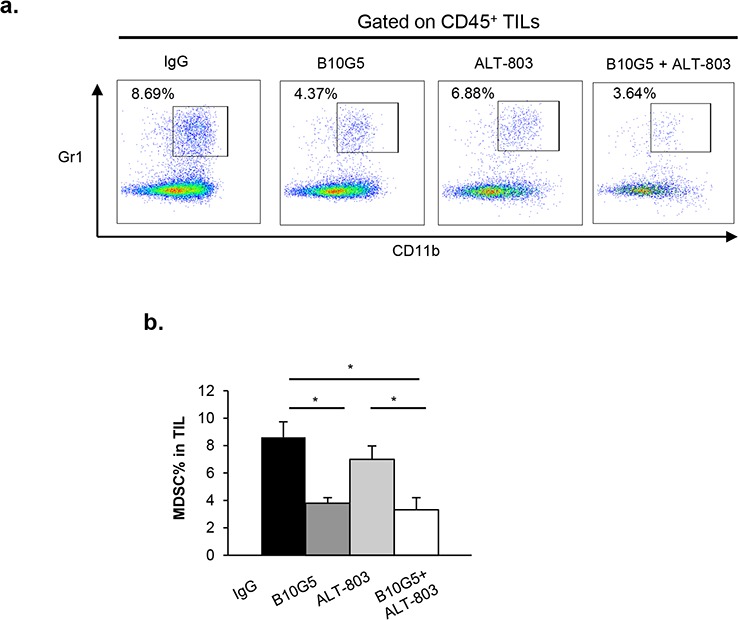
Combination therapy of B10G5 and ALT-803 reduces MDSC population in TILs **a.** Representative dot plots showing percentage of MDSC, defined by CD11b^+^Gr-1^+^, in tumor-infiltrating lymphocytes (TILs) in RM9-sMICB tumors on day 14 of therapy. **b.** Quantification of MDSC percentages in TILs. One grams of tumor from different treatment group were digested to single cell suspension and used for quantifying MDSC percentage. Similar results were obtained from sMIC-B16 tumors. *p < 0.05.

We have recently shown that tumor-derived sMIC can facilitate the expansion of immune suppressive MDSCs, generally defined as CD11b^+^Gr-1^+^ cells [22]. Consistent with our previous findings, neutralizing sMIC significantly reduced the population of MDSCs in RM9-sMICB tumor infiltrates. ALT-803 monotherapy did not significantly impact MDSC population in tumor infiltrates. However, the combination therapy of B10G5 and ALT-803 significantly reduced the MDSC population in tumor infiltrates with a trend of further reduction in comparison to B10G5 monotherapy (Figure 5). We also observed a similar trend toward decreased MDSC frequency in TILs isolated from mice bearing B16-sMICB tumors (data not shown). These results demonstrate that B10G5 can enhance the therapeutic efficacy of ALT-803 through cooperatively eliminating MDSC-mediated immune suppression.

### Combined therapy cooperatively enhances STAT5 signaling

**Figure 6:**
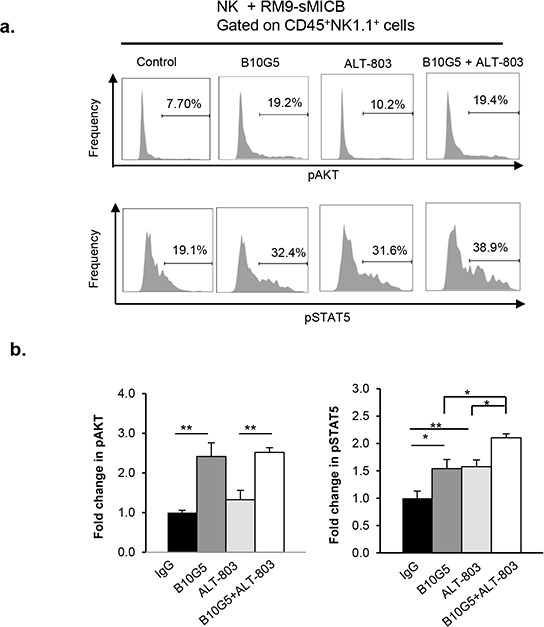
Combined therapy cooperatively sustains STAT5 activation in NK cells in the presence of tumor-derived soluble MIC NK cells derived from spleens of B6/*Rag1^−/−^* mice were co-cultured in a 1:1 ratio with sMICB-RM9 cells in the presence of control mIgG, B10G5 (10 μg/ml), ALT-803 (71 ng/ml), or combination of B10G5 and ALT-803. At 36 h of co-culture, live cells were gated for CD45^+^NK1.1^+^ and analyzed for AKT and STAT5 phosphorylation with intracellular staining and flow cytometry analyses. No IL-2 was present in the co-culture. **a.** Histograms representatively show the phosphorylation status of AKT and STAT5 in NK cells in various culture conditions. **b.** Summary of data from five replicates of four independent experiments. *p < 0.05. **p < 0.01.

To further understand the potential cellular mechanisms whereby combined therapy of B10G5 and ALT-803 enhances NK functionality, purified mouse splenic NK cells were co-cultured with the murine prostate cancer cell line RM9 overexpressing sMICB (RM9-sMICB) in the presence of IgG, B10G5, ALT-803, or the combination of ALT-803 and B10G5. We analyzed the activation status of AKT and STAT5, the two essential molecules for cell survival and effector function. As shown in Figure 6a, the levels of AKT and STAT5 phosphorylation were both increased in the presence of ALT-803 or B10G5. However, the combination of ALT-803 and B10G5 only cooperatively further enhanced STAT5 phosphorylation at a significant level (Figure 6b). Given that STAT5 is essential for NK cell survival and maintaining memory CD8 T cells function, these data suggest that, at the cellular level, the combined therapy of ALT-803 and B10G5 cooperatively enhances NK cell survival and presumably CD8^+^ T cell memory function. Given that AKT is critical for cellular survival and NK cell effector function, our data also suggest that monotherapy of ALT-803 or B10G5 can respectively enhance cellular survival and NK cell functional potential although with a lesser magnitude than the combined therapy.

### Depletion of NK cells eliminates the cooperative effect of B10G5 and ALT-803

**Figure 7:**
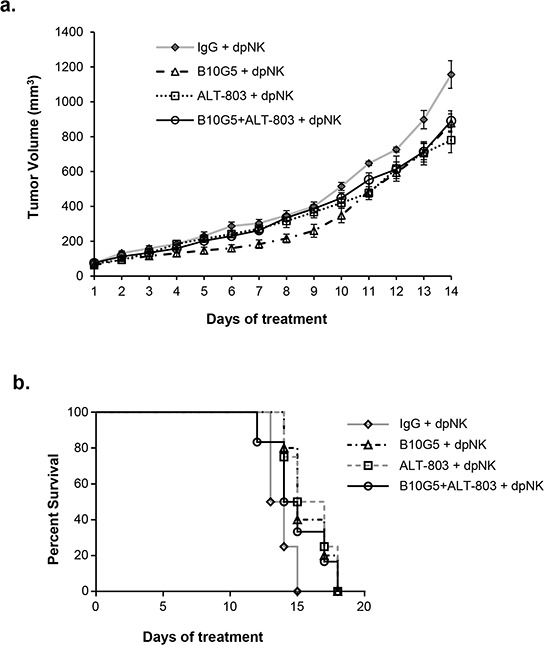
NK depletion impairs the cooperative therapeutic effect of B10G5 and ALT-803 NK cells were depleted by anti-NK1.1 antibody during therapy. **a.** Tumor growth curve. **b.** Kaplan-Meier survive curve. Tumor volume of 1000 mm^3^ was defined as survival endpoint. dpNK, depletion of NK cells.

As both sMIC and IL-15 pose significant effect (negative and positive respectively) on NK cell homeostasis and function, we thus addressed the impact of NK cells in the combined therapeutic effect. As shown in Figure 7, depletion of NK cells abolished the cooperative therapeutic effect of B10G5 and ALT-803, although monotherapy or combined therapy respectively exhibited significant inhibition in tumor growth and prolonged survival. Given that B10G5 or ALT-803 therapy alone has been shown to enhance cytotoxic CD8 T cell function (Figures 3 and 4), the respective therapy effect under the depletion of NK cells is reasonably anticipated.

## DISCUSSION

In the current study, we demonstrated for the first time that the combination therapy of the IL-15 superagonist ALT-803 with a monoclonal antibody B10G5 targeting soluble MIC confers a significant survival benefit in comparison to monotherapy in sMIC^+^ tumor hosts. We showed that the combination therapy not only decreased the rate of tumor growth but also prolonged survival of animals bearing primary tumors in two independent sMIC^+^ syngeneic tumor models. Mechanistically, we further demonstrated that combined therapy cooperatively enhanced homeostasis and function of NK cells, functional potential of effector CD8^+^ and CD4^+^ T cells, and the expansion or sustainability of memory CD8 T cells. We further demonstrated *in vitro* that, in the presence of tumor-derived sMIC, ALT-803 and an antibody neutralizing sMIC cooperatively enhanced STAT5 signaling pathways, which are critical for maintenance of NK cell homeostasis, memory CD8^+^ T cells, and responses of effector CD8^+^ T cells *in vivo* [57–60]. Finally, we demonstrated that NK cells played a significant role in generating the cooperative therapeutic effect.

Human tumor-shed soluble MIC was considered as a viable target for immunotherapy, rationalized by its immune suppressive effect on NK cells and effector T cells and its ability to facilitate the expansion of MDSCs [10, 14, 22]. We have previously shown that MIC neutralization can decrease lung micrometastases of sMIC^+^ tumors and restore NK cell populations and proliferation [10]. In this study, we demonstrated that ALT-803 administration further increased the functional potential of NK, CD4^+^, and CD8^+^ cells in combination with a sMIC-neutralizing antibody. Our findings are consistent with published pre-clinical studies demonstrating the immune stimulatory effect of ALT-803. Xu et al. have shown that in models of murine multiple myeloma, ALT-803 therapy induced expansion of memory CD8^+^ T cells with upregulated NKG2D expression and increased secretion of IFN-γ; in addition, the effect of ALT-803 was shown to be independent of antigen-specific stimulation [45]. Gomes-Giacoia et al. showed that combination therapy of ALT-803 and intravesical Bacillus Calmette-Guerin (BCG) in a rat bladder cancer model resulted in an enhanced therapeutic effect compared to monotherapy. Gomes-Giacoia et al. further demonstrated that the enhanced combinatory therapeutic effect is associated with increased serum and urinary cytokine levels (IL-1α, IL-1β, and RANTES), increased NKG2D^+^ NK populations in the spleen and peripheral blood, and increased NK infiltration into the bladder [61]. These published studies strongly support the underlying mechanisms associated with the enhanced therapeutic effect of ALT-803 and a sMIC-neutralizing antibody as demonstrated in our current study.

Our data demonstrate that combined therapy not only increased NKG2D expression on CD8^+^ T cells but greatly increased the population of CD44^hi^ memory CD8^+^ T cell subset. While the role of IL-15 and ALT-803 in generating and maintaining CD8^+^ memory populations has been well established [38, 45, 62–64], the understanding of NKG2D in memory CD8^+^ T cell homeostasis is limited. Studies by Andre et al. utilizing transgenic mice constitutively expressing the human NKG2D ligand MICA demonstrated that functional NKG2D was dispensable for the generation of memory CD8^+^ responses but necessary for effector functions of reactivated memory cells [65]. Other studies by Zloza et al. demonstrated that NKG2D was important for expansion and maintaining function of memory cells upon reactivation [66]. Zloza et al also demonstrated that IL-15 was crucial for rescue of memory resulting in enhanced cytokine secretion and cytolysis [66]. These limited investigations have suggested that NKG2D is critical for sustaining the immunity of activated memory CD8^+^ T cells. Provided that IL-15 has been well-demonstrated in upregulating NKG2D expression and priming NKG2D signaling pathways, it is reasonable to conclude that the increase in NKG2D function to enhance both effector and memory CD8^+^ T cell function confers a critical mechanism for the cooperative combinatory therapeutic effect.

We show that the combined therapy also cooperatively potentiated CD4 T cells to Th1 responses represented by a significant increase in IFN-γ production in response to re-stimulation, whereas monotherapy only elicited a limited and inconsistent impact in the spleen and dLN. Our observations are consistent with published studies demonstrating that NKG2D^+^CD4^+^ T cells are prone to Th1 cytokine secretion upon co-triggering of IL-15 and NKG2D [67]. Moreover, it has been shown that IL-15 requires IL-12 to cooperatively stimulate Th1 cytokine production by CD4^+^ T cells [68]. In this sense, because activated dendritic cells (DC) in the dLN are the major *in vivo* source for IL-12, it is thus anticipated that ALT-803 may promote Th1 responses by CD4^+^ T cells in the dLN as we have observed. NKG2D is normally absent in CD4^+^ T cells but is expressed by a rare population of effector CD4^+^ T cells in tumor hosts or virally infected individuals [67, 69]. It is an intriguing question how NKG2D signaling alone may regulate potentiation of CD4^+^ T cells to a Th1 response and warrants further investigation.

Tumor-associated MDSCs have been shown to be highly immune suppressive through negatively regulating the function of multiple lymphocyte subsets [70, 71]. MDSCs can inhibit the function of CD8^+^ and dendritic cells though arginase activity and induce the anergy of NK cells through membrane-bound TGFβ1 [72–75]. MDSCs can expand dramatically during tumor progression by tumor-secreted growth factors and cytokines [76, 77]. We have recently shown that tumor-shed sMIC can directly facilitate the expansion of MDSCs through upregulating STAT3 pathways [22]. IL-15 or its agonists have not been shown to play a direct role in regulating MDSC expansion or activity, thus it is anticipated that ALT-803 therapy may only have marginal effect in the population of MDSCs in tumor infiltrates. With B10G5 neutralizing sMIC, we show in this study a significant decrease in the population of MDSCs in tumor infiltrates than ALT-803 treatment alone. As tumor-derived growth factors have been shown to favor the expansion of MDSCs, it is possible that the reduction of MDSC in the tumor infiltrated with the combined therapy is the result of smaller tumor burden. However, since we have shown that neutralizing sMIC reduces the number of MDSC in tumor infiltrates [22], it is also conceivable that the reduction of MDSC may have alleviated its immune suppressive effects on both NK and CD8 T cells and thus cooperatively inhibited tumor growth.

IL-15 can activate several signaling cascades, including the PI3K/AKT/mTOR and STAT5 pathways in effector T and NK cells [78, 79], but the enhancement of signaling in the context of MIC neutralization has not been previously studied. STAT5 has been shown to be essential for NK maturation, peripheral maintenance, and function [57]. Phosphorylation of STAT5 correlates with levels of IL-15 trans-presentation [80]. However, recent studies have shown that IL-15-primed NK cells also require intact PI3K/AKT/mTOR signaling for optimal cytotoxicity, cytokine secretion, and proliferation [81]. These studies suggest that STAT5 and AKT are the two most essential pathways in maintaining the homeostasis and function of NK and memory CD8^+^ T cells. We show that the combination therapy cooperatively enhanced activation of STAT5 pathways and that monotherapy respectively enhanced activation of AKT pathways in NK cells. Although we did not extend our analyses to other cell types, one could anticipate similar outcomes in CD8^+^ T cells. However, it is interesting that the combined therapy only demonstrated limited impact on activating AKT pathways at cellular levels, presumably due to a feedback regulatory mechanism to control normal cellular homeostatic balances.

In summary, given the global immune suppressive nature of human tumor-derived sMIC and the cancer therapeutic potential of the IL-15 superagonist complex ALT-803, which is currently in clinical trials to treat advanced malignancies, our findings provide the rationale for combination therapy of an antibody targeting sMIC and ALT-803 to improve clinical outcomes.

## MATERIALS AND METHODS

### Mice and cell lines

Mice were bred and housed under specific pathogen-free conditions in the animal facility at the Medical University of South Carolina in accordance with institutional guidelines with approved IACUC protocols. All mice used in this study were male rPB-MICB mice on the B6 background as previously described [10]. Transgenic progeny were identified by PCR analysis of DNA extracted from tail biopsies using the forward primer specific for rPB (5′-ACAAGTGCATTTAGCCTCTCCAG TA-3′) and the reverse primer specific for MICB (5′-TGTGTCTTGGTCTTCATGGC-3′).

sMIC-expressing RM9-sMICB and B16F10-sMICB cell lines were developed by transduction with a IRES-GFP retroviral vector containing the construct for recombinant soluble MICB, as described previously [47]. sMIC^+^ cells were selected by puromycin and further by flow cytometry sorting for GFP-positive cells.

### ALT-803 and B10G5 antibody

Generation and characterization of ALT-803 was previously described [48]. Briefly, ALT-803 is a complex consisted of IL-15 with the N72D mutation and the sushi-domain of soluble IL-15R*a* fused to the human Fc region of IgG1. Generation of the anti-MIC mAb B10G5 was also previously described [10]. B10G5 is a non-blocking sMIC-neutralizing monoclonal antibody, which neutralizes free sMIC but does not block the interaction of MIC with the receptor NKG2D [14].

### Tumor inoculation and *in vivo* experiments

RM9-sMICB and B16F10-sMICB cells were implanted subcutaneously into cohorts of syngeneic B6/MICB male mice (*n* = 8–10 per group) (4 × 10^5^ cells/mouse) at ages 8–10 weeks old. When tumor volume reached approximately 75–100 mm^3^, animals were randomized into four therapy groups (*n* = 8–10 per group): 1) control mouse IgG (3.0mg/kg BW); 2) anti-MIC mAb B10G5 (3.0mg/kg BW); 3) ALT-803 (0.2 mg/kg BW); and 4) B10G5 and ALT-803. All therapies were given via I.P. routine twice weekly. In some studies, mice received anti-NK1.1 (PK136, BioXcell) antibody twice weekly via *I.P*. to deplete NK cell during therapy. Tumor volume of 1000 mm^3^ was defined as survival endpoint. After euthanization, spleens, tumor draining lymph nodes and tumors were harvested for analyses.

### *Ex vivo* cytokine re-stimulation and cytotoxicity assay

Single-cell suspensions of splenocytes and tumor draining lymph nodes were stimulated at 37˚C for 6 hr with 50 ng/ml phorbol myristate acetate (PMA) and 500 ng/ml ionomycin. Cells were harvested and analyzed by flow cytometry for intracellular IFN-γ. For cytotoxicity assay, we use CD107a expression as a marker for NK and CD8 T cells degranulation in response to re-stimulation. Specifically, for CD8 T cell function, single cell suspension of bulked splenocytes or tumor draining lymph nodes from B16-sMICB mice was stimulated with 1 μg/ml of melanoma peptide antigen gp100 overnight with addition of PE-labeled anti-CD107a antibody in the culture. For NK cell cytotoxicity assay, NK cells isolated from the spleens were incubated with MHC-I-deficient NKG2D ligand RAE-1β expression RAM-S-RAE-1β cells at 4:1 ratio for 5 h with addition of PE-labeled anti-CD107a antibody in the culture. 1 μM Golgi Plug was added to the culture 4 h before flow cytometry assay.

### *In vitro* co-culture assay

NK cells were generated from spleens of *Rag1−/−* mice as described elsewhere. Briefly, single cell suspensions of splenocytes from *Rag1−/−* mice were seeded at 37°C for 2 h to remove adherent cells. Non-adherent cells were collected and cultured in 1000U/ml of IL-2 for 3–4 days before being harvested for experiments. Cells were > 95% CD3^−^NK1.1^+^ at harvest (data not shown). NK cells were co-cultured with RM9-sMICB cells at 1:1 ratio for up to 36 hours in the presence of control mIgG (10 μg/ml), anti-MICB mAb B10G5 (10 μg/ml), ALT-803 (71 ng/ml), or a combination of B10G5 and ALT-803. Of note, no IL-2 was present in the co-culture. Suspension cells were harvested for analyzing phosphorylation status of pAKT and pSTAT5 in NK cells using flow cytometry by intracellular staining. NK cells were identified by surface CD45 and NK1.1 double positive staining.

### Flow cytometry analysis

Single-cell suspensions were incubated on ice for 30 minutes with a combination of antibodies specific to cell surface markers for identification lymphocyte subsets. These antibodies are: APC- or PE-Cy7-conjugated anti-NK1.1 (clone PK136), APC-Cy7-conjugated anti-CD3ε (clone 145–2C11), FITC-conjugated anti-CD8a (clone 53–6.7), Alexa Fluor 700-conjugated anti-CD4 (clone RM4–5), PE-conjugated anti-NKG2D (clone CX5), PE-conjugated anti-CD44 (clone IM7), PECy7-conjugated anti-CD25 (clone PC61), FITC-conjugated anti-CD11b (clone M1/70), and/or PE-conjugated anti-Gr1 (clone 1A8) (BD Biosciences). For NK cell receptors, fluorochrome-conjugated NKp46, Ly49A, Ly49C/I/F/H, NKG2A/C/E, CD16 were all from eBiosciences. For intracellular staining, cells were stained with surface markers followed by fixation and permeabilization with BD Perm/Fix kits and antibodies specific to intracellular molecules. Fluorochrome-conjugated antibodies specific to IFN-γ, phospho-AKT (pS473), and phospho-STAT5 (pY694) were all from BD Biosciences. Cells were analyzed using the BD Fortessa. Data were analyzed using the FlowJo software (Tree Star).

### Statistics

All statistical data were expressed as mean ± SEM. Difference between means of populations was compared by standard Student's *t* test using One-way ANOVA. Survival was determined via Kaplan-Meier analysis with comparison of curves using the Mantel-Haenszel log-rank test. A *P* value of 0.05 or less was considered significant. GraphPad Prism software was used for all analyses.

## SUPPLEMENTARY FIGURES


