# Supplementary material for: Cooperative therapeutic anti-tumor effect of IL-15 agonist ALT-803 and co-targeting soluble NKG2D ligand sMIC
Source: Oncotarget. 2015 Nov 27;7(1):814–30. doi: 10.18632/oncotarget.6416 (PMC4808035; doi:10.18632/oncotarget.6416)
Supplement: Supplementary file 1 [file oncotarget-07-0814-s001.pdf]

SUPPLEMENTARY FIGURES

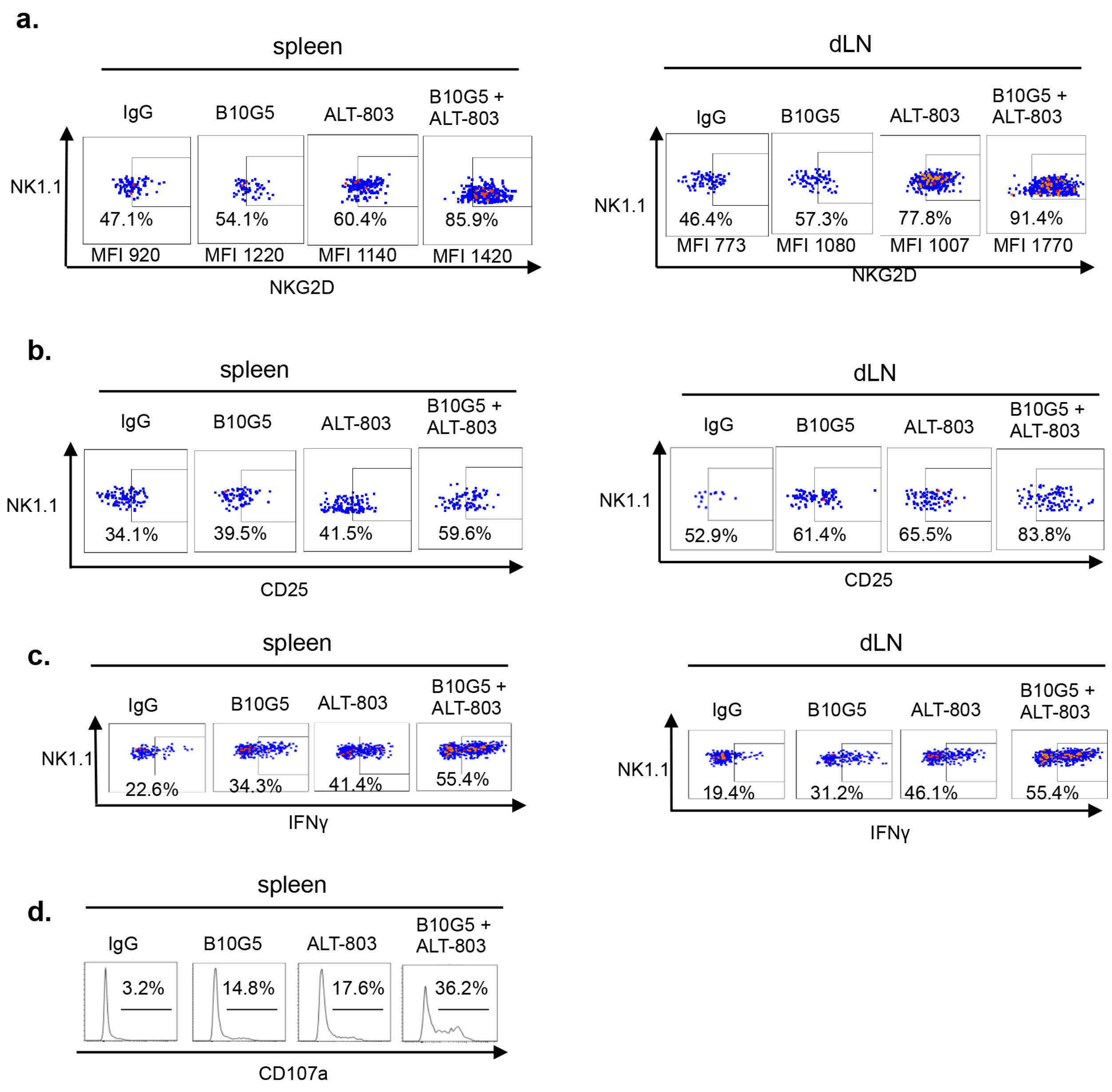

Supplementary Figure S1: Representative dot plots showing coopertaive effect of B10G5 and ALT-803 on NK cell NKG2D expression (percentage of positive cells and MFI of the positive cells) a. activation b. and function c. and d. in the spleen and tumor-draining lymph nodes (dLN) for sMICB-B16 tumor bearing mice in each therapy group. Data were obtained at day 10 since treatment initiation. (a and b) frequencies of surface NKG2D (a) and CD25 (b) expression in $CD3^{-}NK1.1^{+}$ cells. (c) Frequency of NK cells producing IFNγ in response to *ex vivo* PMA/Ionomycin re-stimulation. (d). Degranulation of splenic NK cells shown by CD107a staining when co-cultured with NKG2D ligand expressing RMA-S-RAE-1β cells (3:1 ratio).

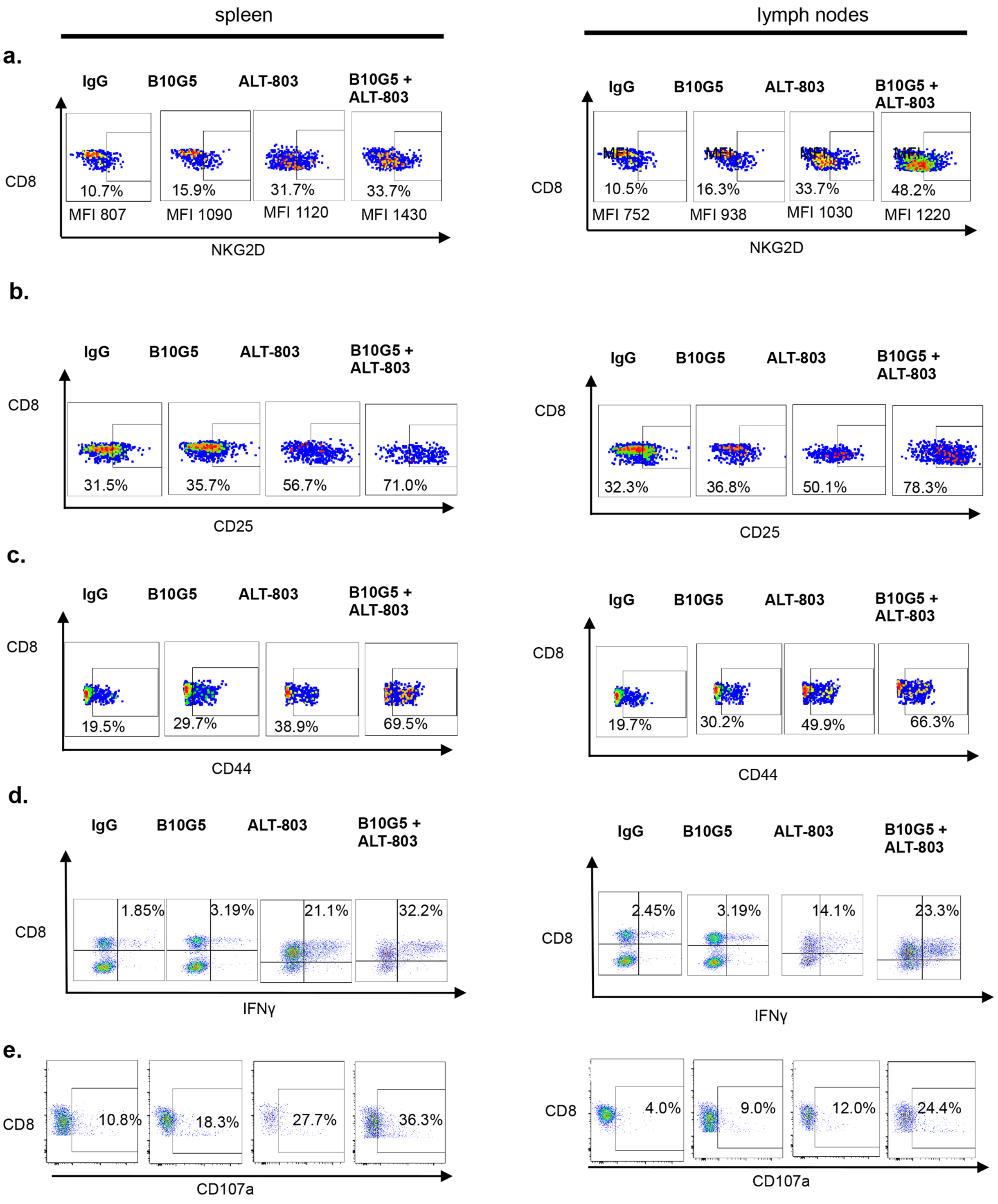

Supplementary Figure S2: Representative dot plots showing the expression of a. surface NKG2D (percentage of positive cells and MFI of the positive cells), b. surface CD25, c. surface CD44, and d. PMA/Ionomycin-induced intracellular IFNγ in CD8 T cells from the spleen and dLN of sMICB-B16 tumor bearing mice in each treatment group. Data were obtained at day 10 since treatment initiation. **e.** Representative dot plots showing CD8 T cell degranulation by CD107a staining in response to *ex vivo* B16 melanoma peptide antigen gp100 stimulation.

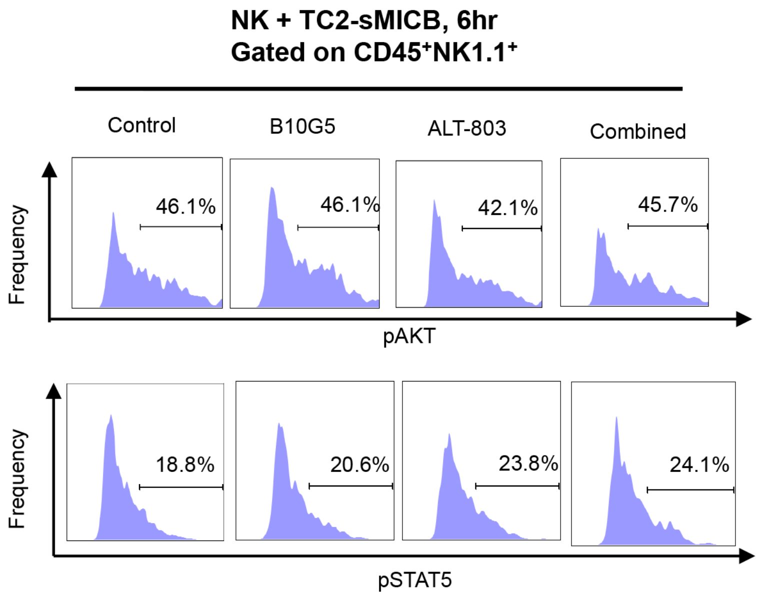

Supplementary Figure S3: NK cells from spleens of *Rag1−/−* mice were co-cultured in a 1:1 ratio with tumor cell lines TC2-sMICB a. or TC2 b. for 12 hours in the presence of control mIgG, B10G5 (10 ug/ml), ALT-803 (71 ng/ml), or combination of B10G5 and ALT-803. Live cells were analyzed via flow cytometry for surface expression of CD45 and NK1.1 and intracellular expression of phosphorylated AKT and phosphorylated STAT5. Representative dot plots showing live cell expression of surface CD45 and NK1.1 and representative histograms demonstrating expression of pAKT and pSTAT5 are shown.
